# Time trends for pulmonary embolism incidence in Greece

**DOI:** 10.1186/s12959-020-0215-7

**Published:** 2020-01-23

**Authors:** Dimitrios G. Raptis, Konstantinos I. Gourgoulianis, Zoe Daniil, Foteini Malli

**Affiliations:** 1 Respiratory Medicine Department, School of Medicine, University of Thessaly, Larissa, Greece; 2 Anatomy and Phsyiology Lab, Nursing Department, University of Thessaly, Larissa, Greece

**Keywords:** Pulmonary embolism, Incidence, Mortality, Epidemiology

## Abstract

**Background:**

Pulmonary embolism (PE) is a disease with a significant impact on public health. However, international epidemiological data are unclear and show considerable heterogeneity. The present study aims to investigate the incidence of PE at the Greek population and the associated demographic characteristics of patients with PE.

**Methods:**

Data on hospital admissions for PE between 1999 and 2012 were provided by the Hellenic Statistical Authority of Greece. Data on age, gender and days of hospitalization from 1999 to 2007 were provided as well. The total population in each region was derived from the 1991, 2001, 2011 Census of the national statistical service of Greece.

**Results:**

The mean annual incidence of PE during the study period was 18.5 per 100.000 population. The annual incidence of PE showed an upward trend ranging from 14 (1999) to 30 (2012) per 100.000 population. In the years before and after the economic crisis faced by Greece we observed statistically significant differences of PE incidence for the two different periods (1999–2008 versus 2009–2012, 14.49 versus 23.06 respectively, *p* = 0.002). The available data revealed a female predominance (16.48 cases for females per 100.000 population versus 13.69 cases for males per 100.000 population, *p* = 0.031). Incidence rate increased with age with a higher incidence in the “80–89” age group.

**Conclusions:**

The incidence of PE appeared to increase in Greece, while it remains below the expected trend in an international context that may be attributed to Computed Tomography Pulmonary Angiography availability and/or PE awareness among clinicians.

## Background

Pulmonary embolism (PE) can be difficult to diagnose and manage. The occurrence of PE is influenced by several factors including aging, cancer and/or hormone replacement therapy [1, 2]. Annual incidence rates of Venous Thromboembolism (VTE) vary significantly and ranges from 62 to 143 per 100,000 persons [3]. Data from United States of America (USA) VTE studies reported that the VTE incidence increased by 82% from 73 to 133 per 100.000 population in the period 1985–2009, that is mainly attributed to an increase in PE [4] and use of Computed Tomography Pulmonary Angiography (CTPA) in the USA [5]. The differentiation may be based on characteristics of the population studied, including age and nationality, on availability of reliable data sources, data from the patients’ medical records only, and on insufficient assessment of primary and recurrent episodes [6].

Data collection on PE hospitalizations is important at national level to evaluate patient outcome and disease incidence. Nevertheless, a small number of studies have examined trends in the incidence of PE during the last two decades. In the USA studies have shown increase in incidence and a significant fall in mortality [5, 7]. Similarly, in the United Kingdom and Australia, rates of admission for PE have increased in recent years [8, 9]. On the other hand, in China, the incidence of PE has remained stable over the past decade, while the mortality rate has decreased [10]. The observed variation between countries may be partly attributed to differences in risk factors for PE or inconsistencies in PE diagnosis between countries.

The data taken together support that PE incidence is increased in recent years, however objective data on the burden of PE are not fully known. To our knowledge there are no available data on PE incidence for the Greek population. In Greece, early reports have shown negative consequences of financial crisis on public health, and notably on respiratory health. The impact of the Greek downturn on respiratory health was obvious although most studies applied data covering only the first years of the crisis and reported its’ short-term outcomes [11].

The aim of the present study was to conduct a nationwide analysis of hospital discharge data of PE, collected from 1999 to 2012. These data were used to elucidate changes in the incidence of patients hospitalized for PE in Greece over a 14-year study period.

## Methods

Information on hospital admissions for PE between 1999 and 2012 were provided by the Hellenic Statistical Authority of Greece. The Hellenic Statistical Authority (ELSTAT) is an independent organization enjoying operational, administrative and financial independence that coordinates the functions of the other agencies in the Hellenic Statistical System. Its’ operation is subject to the control of the Hellenic Parliament but not to the control of governmental bodies or other administrative authority. ELSTAT coordinates all the agencies that have the responsibility or obligation to collect the country’s official statistics and forwards these statistics to Eurostat. The services and agencies of the public sector, the Legal Entities under Private Law, the associations of individuals and natural persons are obliged to grant ELSTAT access to all the administrative sources, public registers and files they keep, in printed, electronic or other form, and provide, in an accurate and timely manner, ELSTAT with primary statistical data and information, which is required for the performance of its duties. The data provided to ELSTAT from government entities and administrative sources are subjected to controls by ELSTAT with a view to assessing their accuracy and reliability before being used in the production of statistics by Hellenic Statistical Authority. The mission of the Hellenic Statistical Authority is to safeguard and continuously improve the quality of the country’s statistics by following in all areas the highest European and international standards of statistical practice, as well as by unswervingly observing the rules and responsibilities it is committed to [12]. Data on age, gender and days of hospitalization from 1999 to 2007 were provided as well. The dataset of 1999 to 2007 contained data on deaths where PE was reported as a cause of death in the death certificate. The annual incidence of PE was estimated as the number of hospital discharges with PE diagnosis in 1 yr (including fatal cases of PE) to the total population and expressed as the number of events per 100.000 population. The discharges were recorded using the ICD 9 and ICD 10 system, depending on the system in force at the time of each discharge. Specifically, the calculation of PE incidence resulted from the quotient of new cases (discharges) during a comparable year in a region to the total population in the same year in that region expressed per 100.000 population. Briefly ICD 10 codes used for Pulmonary embolism cases were I26 (I26.0 and I26.9) and ICD 9 codes were 415.1. The total population in each region was derived from the 1991, 2001, 2011 Census of the national statistical service of Greece [12].

The incidence of PE (per 100.000 population) was calculated for each year from 1999 to 2012 in each of the 10 regional areas of Greece and in the total population of Greece. In addition, these 14 years were divided into two groups with a milestone in 2008 which presents the start of the economic crisis. In detail, two groups were created, a group of 1999–2008 and one of 2009–2012 in the average incidence wad calculated. For the processing of age, we used ten-year intervals from the age of 10 up to the age of 100 and over, and the outcome was 10 age-categories.

Data on all patients identifiers were not provided to the authors in order to assure patient confidentiality. The study protocol was approved by the ethics committee of our institution.

### Statistical analysis

Demographic characteristics are reported as mean ± standard deviation unless otherwise indicated. All datasets were tested for normality using the Shapiro-Wilk normality test. Incidence rates comparison was performed using a parametric t-test.

All the statistical analysis was performed at the statistical significance level of 5% corresponding to *p* value of 0.05. Data were analyzed using SPSS software, version 22 (Statistical Package for Social Sciences Inc., 2003, Chicago, USA).

## Results

**Fig. 1:**
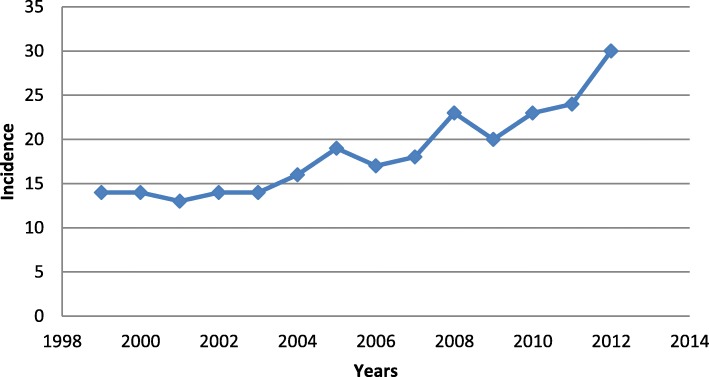
Annual incidence of pulmonary embolism among residents of Greece, from 1999 to 2012

The average annual incidence of PE in Greece for the 14 years of our study was 18.5 per 100.000 (95% CI: 15.61–21.39). We observed an upward trend in PE incidence during the period 1999 to 2012. During this period a total of 27.347 cases of PE in Greece were reported to the National Statistical Service of Greece, with incidence per 100.000 population ranging from 13 to 30 per year (Fig. 1). PE showed a higher incidence in 2012 when compared to previous years, with an average annual reported incidence of 30 outbreaks per 100.000 population (number of cases: 3096). Table 1 presents PE incidence in Greece per geographical area in the years before and after the economic crisis faced by Greece (at 2008). We observed statistically significant differences of PE incidence for the two different periods of time (1999–2008 versus 2009–2012, 14.49 versus 23.06, respectively, *p* = 0.002). When comparing the 10 regional areas with the intervals 1999–2008 and 2009–2012, we noticed that there are areas with a large increase in incidence, while only one remained approximately at the same level (Additional file 1: Table S1 and Figure S1A and S1B). Specifically, we observed that Thessaly and the Ionian Islands have the largest increase in the incidence, Epirus has a small increase, while Macedonia has a very small decrease (*p* < 0.001).

**Table 1:** Incidence of PE per geographic area 2 time periods, 1999–2008 (before the economic crisis) and 2009–2012 (following the economic crisis). The values correspond to cases per 100.000 population. ^#^p = 0.002

		1999–2008	2009–2012
Incidence rate	Attica	16	27
	Central Greece	9.38	16.07
	Peloponnese	12.51	17.22
	Ionian Islands	11	25
	Epirus	20.83	25.04
	Thessaly	11	32
	Macedonia	22.01	20.74
	Thrace	14	27
	Aegean Islands	11.2	16.52
	Crete	17	24
	Total Greece	14.49	23.06^*#*^

**Fig. 2:**
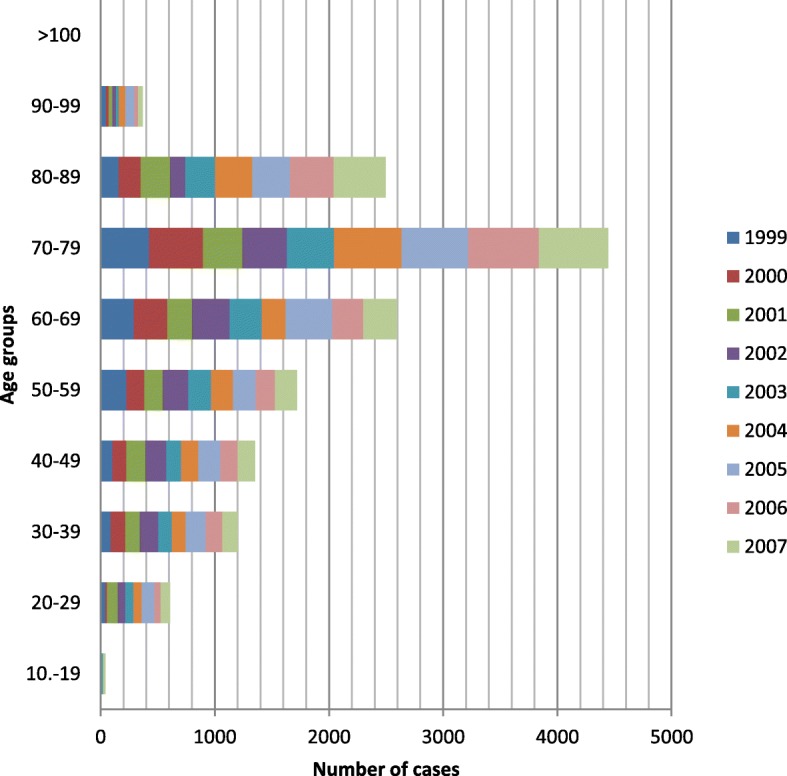
Age distribution of PE from 1999 to 2007

Incidence rate increases with age. Figure 2 presents the age distribution of PE from 1999 to 2007 where available data concerning age where provided. The number of cases reported during this period was 14.827 and showed an increasing trend from the age group “10–19” to “70–79”, however, there is a downward trend after the age of 79. In more details, the disease showed a higher proportion in the “70–79” age group with approximately 30% (*n* = 4446) of cases, followed by “60–69” and “80–89” age groups with 17.50% (*n* = 2595) and 16.84% (*n* = 2497) of total cases respectively. As for the age groups “50–59”, “40–49”, “30–39” and “20–29” the proportion was 11.60% (*n* = 1720), 9.11% (*n* = 1352), 8.10% (*n* = 1201) and 4.10% (*n* = 608) of cases respectively. As expected, the age group with the fewest cases of PE was “10–19” with a percentage of 0.26% (*n* = 40).

**Fig. 3:**
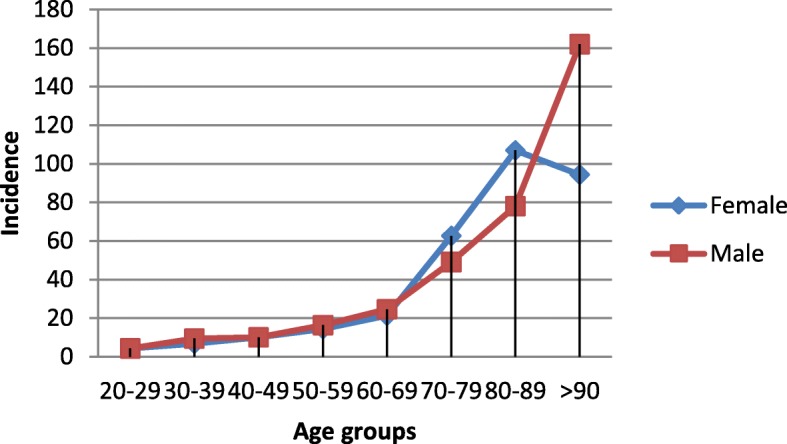
Average PE incidence of 1999–2007 per age group for females and males

The distribution of PE cases from 1999 to 2007 by sex is presented in Fig. 3. From the total number of patients examined during this period, 44.9% (*n* = 6668) of cases were males and 55.1% (*n* = 8191) were females. We observed a female predominance that was not present in all years studied (Additional file 1: Figure S2). Table 2presents annual incidence of PE from 1999 to 2007 sorted by age and sex. The mean incidence increases with age for both genders with a peak at the age group of 80–89 for females and > 90 for males. Corresponding graphs are presented as Additional file 1: (Figure S3A and Figure S3B).

**Table 2:** Annual age-adjusted incidence of PE cases during the years 1999 to 2007 for females and males as distributed by age. Mean incidence corresponds to mean annual incidence per age group throughout the years studied

Age	Female												Male											
	Population*	1999	2000	2001	2002	2003	2004	2005	2006	2007	Mean incidence	95% CΙ	Population*	1999	2000	2001	2002	2003	2004	2005	2006	2007	Mean incidence	95% CΙ
10–19	626,778	0	0	0	0	2,55	0	0	0	1,28	0,4256	-0,2679-11,190	686,037	1,17	0	0	0	0	0	0	0	1,17	0,2600	-0,1366-0,6566
20–29	809,332	0,99	0,99	6,92	3,95	4,94	5,93	5,93	3,95	3,95	41,722	25,709-57,736	872,420	3,67	0,92	4,58	3,67	3,67	2,75	7,34	6,42	5,5	42,800	27,848-57,752
30–39	819,475	5,86	7,81	6,83	8,79	3,9	4,88	7,81	8,79	5,86	67,256	54,004-80,507	833,355	4,8	7,68	8,64	10,68	10,56	9,6	13,44	8,64	10,56	94,000	75,575-11,2425
40–49	752,130	7,45	9,57	10,64	14,89	5,32	11,7	11,7	8,51	10,64	10,0467	79,189-12,1745	743,368	6,46	6,46	11,84	9,69	11,84	8,61	14	11,84	9,69	10,0478	80,633-12,0323
50–59	638,375	15,04	16,29	10,03	17,54	12,53	12,53	16,29	16,29	12,53	14,3411	12,4091-16,2732	608,950	21,02	9,2	15,76	18,39	19,71	18,39	15,76	10,51	18,39	16,3478	13,2313-19,4643
60–69	673,592	13,21	24,94	19	27,32	17,96	16,63	32,07	20,19	20,19	21,2789	16,7997-25,7580	589,611	34,09	21,71	14,93	24,42	27,14	16,28	32,56	23,07	27,14	24,5933	19,5838-29,6028
70–79	482,051	61,4	59,74	49,79	54,77	46,88	76,55	69,7	84,85	59,95	62,6256	53,0973-72,1538	391,770	32,67	47,22	26,55	32,67	46,97	57,18	63,3	53,09	81,68	49,0367	35,7209-62,3524
80–89	168,725	75,86	56,9	118,54	56,9	85,35	137,5	104,31	170,69	156,47	106,9467	74,7534-139,1400	124,548	25,69	77,08	44,96	32,12	90,73	77,08	122,04	77,08	154,16	77,8822	45,9139-109,8505
> 90	27,340	87,78	87,78	58,52	87,78	0	146,31	175,57	117,04	87,78	94,2844	55,6883-132,8806	9337	257,04	0	171,36	85,68	257,04	171,36	342,72	0	171,36	161,8400	71,9928-251,6872
Total	4,997,798	13,49	14,93	15,22	16,23	12,66	18,28	19,56	20,6	17,41	16,4867	14,4143-18,5591	4,859,396	11,85	10,81	10,35	11,55	14,95	13,16	18,48	13,31	18,77	13,6922	11,2893-16,0951

*According to the 2001 census

**Total Greek population with age group “0–9”: 10932136

The total incidence of PE for the years 1999 to 2007 for females was estimated at 16.48 cases per 100.000 population and for males at 13.69 cases per 100.000 population (*p* = 0.031, Table 2 and Fig. 3). We observed a female predominance in PE age-adjusted incidence for the age-groups of 70–79 and 80–89 and a male predominance for the age groups 10–19, 20–29,30–39, 40–49, 50–59, 60–69 and > 90 (Table 2).

**Fig. 4:**
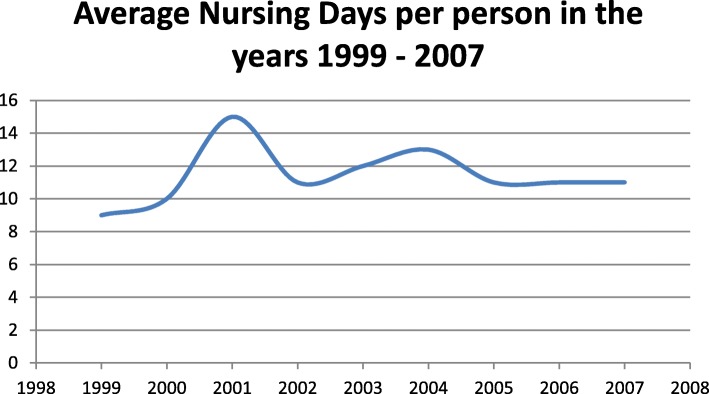
Average Nursing Days per person in the years 1999–2007

Figure 4 shows the variation in the days of hospitalization per person in the years 1999 to 2007. Average nursing days in the study period are 11.44 ± 1.74. We observed that nursing days are approximately constant throughout the years.

**Fig. 5:**
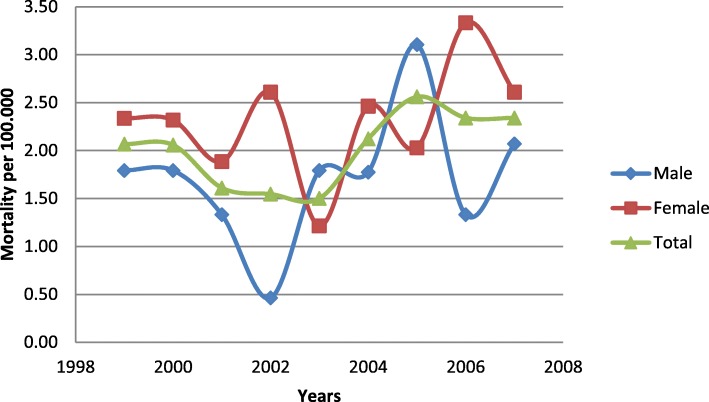
Mortality rates from PE

Data on mortality were provided for the years 1999 to 2007 and are shown in detail in Additional file 1: Table S1. Mortality rate for the studied period was 2.01 ± 0.38 (95% CI: 1.72–2.31) per 100.000 population. We observed an increase in mortality rates from 1999 to 2007 although not statistically significant (Fig. 5).

## Discussion

To our knowledge, this is the first study addressing PE incidence in the Greek population. According to our results, PE incidence is 30 cases per 100.000 population for 2012 that seems to be lower than previously published reports from other countries [3, 4]. Additionally, we demonstrated that there is an upward trend in PE incidence from 1999 to 2012, probably reflecting advances in available diagnostic tests among others. The available data revealed a female predominance that was mainly attributed to the age group of > 70 years. As expected, PE incidence rised with older age with a peak at years 80–89.

The annual incidence in PE for 2012 was estimated to 30 per 100.000 population. The reported incidence in PE is lower than previously reported by others. Dentali et al. [13] observed an incidence of 55.4 for females and 40.6 for males per 100.000 population in Northwestern Italy. Date from the USA report an incidence of VTE of 133 cases per 100.000 population [4]. The significant variation may be attributed to multiple factors including age distribution of the population studied, ethnicity, CTPA availability and/or PE awareness among clinicians [6]. The design of our study did not address this data and further studies are required to elicit the significant variation of PE incidence among studies.

We observed annual increases in PE incidence in the 14 years studied. We demonstrated a PE incidence of 30 cases per 100.000 population in 2012 and 14 cases per 100.000 population in 1999. The increase in PE incidence was evident in both genders. Our results are in agreement with previously published data [5, 8, 13–16] suggesting an upward trend in PE event rates throughout the years. The observed trends may be partially explained by the continuous improvement in diagnostic strategies for the identification of the disease. The wide availability of CTPA along with the greater adoption of diagnostic algorithms may explain the rise in PE incidence. A greater awareness of PE among clinicians and the higher rate of incidental diagnosis of PE (when Computed Tomography was performed for other reasons, i.e. cancer staging) may to some extent account for the increased rates of PE [9].

Our results provide further support to the age-dependent increase in VTE risk. We have observed increased PE incidence in older subjects with a peak at the age groups > 80 years for both genders. Our findings are consistent with previously published data. Incidence rates of PE in elderly patients are three times as high when compared to younger patients [17]. The factors underlying the increase in VTE risk with age are multiple and include alterations in coagulation system proteins, platelet activity and inflammatory state among others [18].

Our data revealed a female predominance in PE incidence that was mainly attributed to the age groups of 70–79 and 80–89 years with no significant differences in age-adjusted incidence in the younger age groups for both genders. Traditionally the age-adjusted incidence of PE is considered higher among males with a male to female ratio of 1.2:1. Studied have previously shown a female predominance in PE incidence [13] although age-adjusted incidence of VTE among males and females presents no differences suggesting that sex does not significantly impact on VTE incident cases [13, 19]. We observed significant differences in total PE incidence among females and males. One possible explanation could be differences in life expectancy amongst sexes. The life expectancy of females is greater than males for the study period (ranging from 81.1 in 1999 to 83.4 years in 2012 for females and 75.90 in 1999 to 78 years in 2012 for males) [20]. Additionally, differences in thrombotic and fibrinolytic activity between the two sexes may implicate the sex-related discrepancies of PE incidence in older age [21]. The factors, contributing to the sex dependent increase in PE incidence in our population merits further research in future studies.

We have demonstrated a rather low rate of PE related deaths although the mortality rate seems to increase from 1999 to 2007. The mortality rate reported in our study is lower than the one reported in United Kingdom [8] and USA [7] and approximately the same with the one reported in Australia [9]. Additionally mortality rates increase over time in certain countries and decrease in others [22]. The reasons for the increase in PE related deaths throughout the year are uncertain and cannot be addressed with the data available in our study.

It is well known that the financial crisis in Greece has significantly reduced health expenses from 13.2% in 2006 to 11.5% in 2012 [23, 24]. Studies have demonstrated a deterioration of self-rated health during the economic crisis [25, 26], while the financial austerity has been associated with an increase in people reporting unmet medical needs [27]. Greece has ranked 4th out of 30 countries in terms of deaths from the A(H1N1) influenza virus and the Western Nile virus outbreak during the period 2009–2012 [28]. However, austerity has been associated with important positive steps including the standardization of the health benefits package for all citizens and new monitoring tools for hospital management and the development of e-health governance tools. We demonstrated a statistically significant increase in PE incidence between two time frames, 1999–2008 and 2009–2012 that corresponds to the economic crisis. We attribute the increase in PE diagnosis to the improvements of diagnostic tests and possibly to the increased awareness for the disease as well as the rise in the use of public services as opposed to private ones [29, 30]. Unfortunately, our study was not designed to address this question and no definite conclusions can be drawn regarding the explanation of our findings.

Our study has several strengths and limitations. This is the first report of PE incidence in Greece and covers data for a long period of time (14 years). However we acknowledge that data on age, sex, days of hospitalization and mortality are limited to the years 1999–2007 since the Hellenic Statistical Authority had no available data thereafter. Unfortunately, the reasons that underlie this are not available to us. One possible explanation could be that the economic downturn faced by Greece could influence the department(s) resourcing and that may have influenced the data reporting. To our knowledge there was no reduction in the personnel responsible for coding, although this merits further exploration. Additionally, our study is of retrospective nature while we did not have available data on demographics of the cases (besides age and gender) or VTE related risk factors like cancer or hormone-replacement therapy. Our study was based on the discharge diagnoses (including fatal cases) of PE from all the Greek provinces during the years 1999 and 2012. The Hellenic Statistical Authority provided data on gender and age distribution of PE diagnosis on each geographical department. Unfortunately, data on the risk factors associated with the reported cases (including comorbidities, hospitalization status, use of thromboprophylaxis, etc) were not available to us. We have included only PE cases that were recorded by hospital discharges irrespective of length of hopsitalization (including early discharfe or < 24 h hopsital stay). PE cases managed as outpatients were not included in the analysis. However, we have reason to believe that the proportion of patients managed as outpatients would be rather small. Analysis form the RIETE registry suggests that only a small proportion of PE patients are managed as outpatients for our study period (ranging from 0.03 to 1.7% for 2001 to 2013, respectively) suggesting a minimal estimation bias [31].

## Conclusions

In conclusion, the results of our study confirm a not negligible incidence of PE in the Greek population. During the 14 years of observation the incidence of PE appeared to increase, while it remains below the expected trend in an international context. The frequency of CTPA testing has increased in emergency departments, and we suggest that further studies could analyze national data to determine how increased rates of diagnosis for PE compare with the utilization of outpatient management options for low-risk PE.

## Supplementary information

**Additional file 1 **: **Figure S1** Incidence of pulmonary embolism in Greece from 1999 to 2008 (A) and 2009–2012 (B). **Figure S2** Distribution of PE between the two genders during the periods 1999 to 2007. **Figure S3** Percentage of PE cases in females (A) and males (B) throughout the years 1999 to 2007. **Table S1** Number of deaths and Mortality from PE during the years 1999–2007

## Data Availability

The datasets generated and/or analysed during the current study are available in the Hellenic Statistical Authority of Greece [www.statistics.gr].

## Abbreviations

CTPA: Computed Tomography Pulmonary Angiography
PE: Pulmonary Embolism
SPSS: Statistical Package for Social Sciences
USA: United States of America
VTE: Venous Thromboembolism

## References

[1] Goldhaber SZ (2004). Pulmonary embolism. Lancet.

[2] Kyrle PA, Eichinger S (2005). Lancet.

[3] Spencer FA, Emery C, Joffe SW (2009). Incidence rates, clinical profile, and outcomes of patients with venous thromboembolism. The Worcester VTE study. J Thromb Thrombolysis.

[4] Huang W, Goldberg RJ, Anderson FA, Kiefe CI, Spencer FA (2014). Secular trends in occurrence of acute venous thromboembolism: the Worcester VTE study (1985-2009). Am J Med.

[5] Wiener RS, Schwartz LM, Woloshin S (2011). Time trends in pulmonary embolism in the United States: evidence of overdiagnosis. Arch Intern Med.

[6] Cushman M (2007). Epidemiology and risk factors for venous thrombosis. SeminHematol.

[7] Horlander KT, Mannino DM, Leeper KV (2003). Pulmonary embolism mortality in the United States, 1979-1998: an analysis using multiple-cause mortality data. Arch Intern Med.

[8] Aylin P, Bottle A, Kirkwood G, Bell D (2008). Trends in hospital admissions for pulmonary embolism in England: 1996/7 to 2005/6. ClinΜed.

[9] Shiraev TP, Omari A, Rushworth RL (2013). Trends in pulmonary embolism morbidity and mortality in Australia. Thromb Res.

[10] Yang Y, Liang L, Zhai Z (2011). Pulmonary embolism incidence and fatality trends in Chinese hospitals from 1997 to 2008: a multicenter registration study. PLoS One.

[11] Kotsiou OS, Zouridis S, Kosmopoulos M, Gourgoulianis KI (2018). Impact of the financial crisis on COPD burden: Greece as a case study. Eur Respir Rev.

[12] Hellenic Statistical Authority of Greece. [www.statistics.gr]. Access 16/12/2019.

[13] Ageno Walter, Pomero Fulvio, Fenoglio Luigi, Squizzato Alessandro, Bonzini Matteo, Dentali Francesco (2016). Time trends and case fatality rate of in-hospital treated pulmonary embolism during 11 years of observation in Northwestern Italy. Thrombosis and Haemostasis.

[14] Park B, Messina L, Dargon P, Huang W, Ciocca R, Anderson FA (2009). Recent trends in clinical outcomes and resource utilization for pulmonary embolism in the United States: findings from the nationwide inpatient sample. Chest.

[15] de Miguel-Díez J, Jiménez-García R, Jiménez D (2014). Trends in hospital admissions for pulmonary embolism in Spain from 2002 to 2011. Eur Respir J.

[16] Mellemkjaer L, Sorensen HT, Dreyer L, Olsen J, Olsen JH (1999). Admission for and mortality from primary venous thromboembolismin women of fertile age in Denmark, 1977–95. BMJ.

[17] Cushman M, Tsai AW, White RH (2004). Deep vein thrombosis and pulmonary embolism in two cohorts: the longitudinal investigation of thromboembolism etiology. Am J Med.

[18] Wilkerson WR, Sane DC (2002). Aging and thrombosis. InSeminars in thrombosis and hemostasis.

[19] Tagalakis V, Patenaude V, Kahn SR, Suissa S (2013). Incidence of and mortality from venous thromboembolism in a real-world population: the Q-VTE study cohort. Am J Med.

[20] Countryeconomy.com [https://countryeconomy.com/demography/life-expectancy/Greece], Access 16/12/2019.

[21] Stegnar M, Pentek M (1993). Fibrinolytic response to venous occlusion in healthy subjects: relationship to age, gender, body weight, blood lipids and insulin. Thromb Res.

[22] Hoffmann B, Gross CR, Jöckel KH, Kröger K (2010). Trends in mortality of pulmonary embolism–an international comparison. Thromb Res.

[23] OECD (2013), Health at a Glance 2013: OECD Indicators, OECD Publishing. 10.1787/health_glance-2013-en.

[24] WHO Regional Office for Europe (2014). Health for All database [online/ offline database]. Copenhagen, WHO Regional Office For Europe (http://data.euro.who.int/hfadb, accessed 2 November 2014).

[25] Kentikelenis A, Karanikolos M, Papanicolas I, Basu S, McKee M, Stuckler D (2011). Health effects of financial crisis: omens of a Greek tragedy. Lancet.

[26] Zavras D, Tsiantou V, Pav E, Mylona K, Kyriopoulos J (2013). Impact of economic crisis and other demographic and socio-economic factors on self-rated health in Greece. Eur J Pub Health.

[27] Economou C, Kaitelidou D, Kentikelenis A, Maresso A, Sissouras A. The impact of the crisis on the health system and health in Greece. Economic crisis, health systems and health in Europe: Country experience [Internet]. EuropeanObservatoryonHealthSystemsandPolicies, 2015.

[28] Bonovas S, Nikolopoulos G (2012). High-burden epidemics in Greece in the era of economic crisis. Early signs of a public health tragedy. J Prev Med Hyg.

[29] Ministry of Health and Social Solidarity. ESYnet database. Athens, 2012a.

[30] Ministry of Health and Social Solidarity. Ministry of Health and Social Solidarity. Report on the outcomes of Ministry of Health and its health units, 2011. Athens, Dionikos, March 2012 [inGreek], 2012b.

[31] Jiménez D, de Miguel-Díez J, Guijarro R (2016). Trends in the management and outcomes of acute pulmonary embolism: analysis from the RIETE registry. J Am Coll Cardiol.

