# Supplementary material for: Time trends for pulmonary embolism incidence in Greece
Source: Thromb J. 2020 Jan 23;18:1. doi: 10.1186/s12959-020-0215-7 (PMC6979064; doi:10.1186/s12959-020-0215-7)
Supplement: Supplementary file 1 — Additional file 1 : Figure S1 Incidence of pulmonary embolism in Greece from 1999 to 2008 (A) and 2009–2012 (B). Figure S2 Distribution of PE between the two genders during the periods 1999 to 2007. Figure S3 Percentage of PE cases in females (A) and males (B) throughout the years 1999 to 2007. Table S1 Number of deaths and Mortality from PE during the years 1999–2007 [file 12959_2020_215_MOESM1_ESM.docx]

Figure S1

*Incidence of pulmonary embolism in Greece from 1999 to 2008 (A) and 2009-2012 (B)*


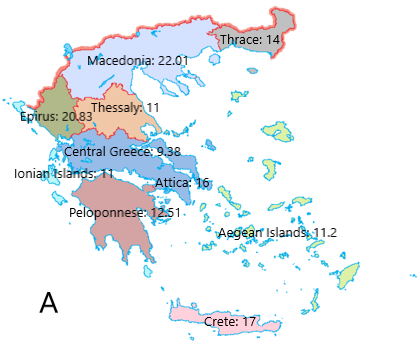


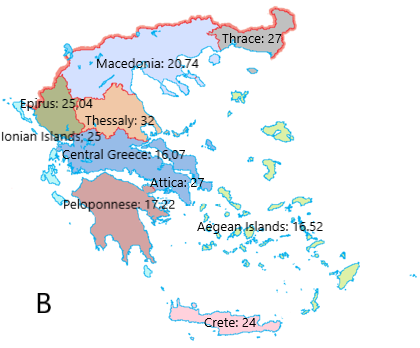


Figure S2

*Distribution of PE between the two genders during the periods 1999 to 2007.*

-

Figure S3

*Percentage of PE cases in females (A) and males (B) throughout the years 1999 to 2007*

Table S1

Number of deaths and Mortality from PE during the years 1999-2007

| **Number of Deaths** | | | | **Mortality** | | |
| --- | --- | --- | --- | --- | --- | --- |
| **Year** | **Male** | **Female** | **Total** | **Male** | **Female** | **Total** |
| 1999 | 97 | 129 | 226 | 1,79 | 2,34 | 2,07 |
| 2000 | 97 | 128 | 225 | 1,79 | 2,32 | 2,06 |
| 2001 | 72 | 104 | 176 | 1,33 | 1,88 | 1,61 |
| 2002 | 25 | 144 | 169 | 0,46 | 2,61 | 1,55 |
| 2003 | 97 | 67 | 164 | 1,79 | 1,21 | 1,50 |
| 2004 | 96 | 136 | 232 | 1,77 | 2,46 | 2,12 |
| 2005 | 168 | 112 | 280 | 3,10 | 2,03 | 2,56 |
| 2006 | 72 | 184 | 256 | 1,33 | 3,33 | 2,34 |
| 2007 | 112 | 144 | 256 | 2,07 | 2,61 | 2,34 |
